# Incidence of lameness and abrasions in piglets in identical farrowing pens with four different types of floor

**DOI:** 10.1186/1751-0147-51-23

**Published:** 2009-05-23

**Authors:** Mate Zoric, Ebba Nilsson, Nils Lundeheim, Per Wallgren

**Affiliations:** 1 National Veterinary Institute, SVA, 751 89 Uppsala, Sweden; 2 Department of Clinical Sciences, Swedish University of Agricultural Sciences, SLU, Box 7054, 750 07 Uppsala, Sweden; 3 Department of Animal Breeding and Genetics, Swedish University of Agricultural Sciences, Box 7023, 750 07 Uppsala, Sweden

## Abstract

**Background:**

Lameness in piglets is a major animal welfare issue. Floor abrasiveness is a common cause of superficial injury in piglets in farrowing pens. The abrasion achieved may act as a gate for infections, which in turn may induce development of infectious arthritis. In this study, the influence of improvements of the floor quality and of increased ratios of straw in identical farrowing pens was measured.

**Methods:**

The study was carried out at a herd with four identical farrowing units with solid concrete floor bedded with 1 kg chopped straw per sow and 1 hg per piglet and day. Nothing was changed in the management of the four identical farrowing units, but four experimental groups were created: Group I – control, Group II – the amount of bedding was doubled. The surface of the floor was repaired in two units, Group III – Piglet Floor^®^, Flowcrete Sweden AB, Perstorp, Sweden and Group IV – Thorocrete SL^®^, Växa Halland, Sweden. Three farrowing batches were studies in each unit. In total, 93 litters (1,073 piglets) were examined for foot and skin lesions until the age of 3 weeks. The occurrence of lameness was registered until weaning at an average age of 4.5 weeks. Twenty seven lame piglets were culled instead of medicinally treated and subjected to necropsy including histopathological and microbiological examinations. Isolates of streptococci, staphylococci and *E. coli *were tested with respect to antimicrobial resistance.

**Results:**

Piglet born on the repaired floors had the lowest prevalences of abrasions at carpus. Also the doubled straw ration decreased the abrasions. Skin lesions at carpus decreased significantly in magnitude in all four systems from day 10. At day 3, the sole bruising scores of the control unit were greater than the other three units (p < 0.001). At day 10 and 17, sole bruising was less common in the units with repaired floors than in the control group and the group with doubled straw ration. In total 41 piglets were diagnosed as lame, corresponding to 3.8% of all live-born piglets (n = 1,073). Around 85% of these diagnoses took place during the first 3 weeks of life and the risk incidence of lameness decreased from 1.5% during the first week of life to 0.5% during the fourth week. The incidence of lameness was highest in the control unit and lowest in the units with repaired floors. Twenty lame piglets were confirmed to have bacterial growth in the joint. The causative agents were *Streptococcus dysgalactiae *subsp. equisimilis (60%), Staphylococcus hyicus subsp. hyicus (35%) and Escherichia coli (5%). These isolates were sensitive to all antibiotics included in the antimicrobial panels.

**Conclusion:**

The results suggest that proper maintenance of the floor can prevent the degree of roughness and abrasiveness of the floors, which in turn can contribute significantly to prevention of abrasions, sole bruising and lameness in piglets. Maintaining the surface of concrete floors with two different commercially available solutions both decreased the incidence of abrasions and sole bruisings and thereby also of arthritis significantly. Also doubling the amount of chopped straw turned out to prevent development of skin lesions and sole bruisings to some extent, and subsequently also the incidence of arthritis.

## Background

Due to roughness of the surface in floors of farrowing pens, newborn piglets may develop foot and skin lesions during their first days of life. The abrasion injuries that are most severe on the front legs [1-4] are the result of contact with the floor and they may be amplified by paddling at suckling [2]. Straw or other litter in the pen is not always effective in preventing this, because the piglets may remove the litter with their physical activity. Besides, use of straw is limited in modern pig production for of economical reasons such as harvesting, labour and interference with dung removal [5].

Rough and abrasive floors may harm horn and skin of the feet of newly born piglets, causing acute lameness due to pain. The frequency of skin lesions increases significantly over the first 3 days of life [3,4,6], and if the floor is hard and rough the healing process may be delayed. This is of importance because the abrasions can be entry points for infections, which in turn may lead to ill-thrift in terms of lameness caused by arthritis and also to a decrease in productivity [7,8].

During the first week of life the piglets spend most of their time either lying in the nest or suckling. If the floor on these spots is hard and rough skin lesions are likely to develop. Straw or other litter in the pen is not always effective in preventing this, because the piglets remove the litter out of the lying area during physical activity [9], and it is obvious that the immediate environment of the piglets plays a primary etiological role in the appearance and development of leg injuries in suckling piglets. However, the provoking factors may vary between herds. They include the design of the farrowing pen, the construction and condition of the floor surface as well as type and amount of bedding material used [10].

The impact of lameness may by very costly [11], but it can be reduced by treatment of affected piglets and by a correct maintenance of the floor surfaces [12]. In this study, we measured the influence of the floor quality and of increased ratios of straw in identically farrowing pens managed by the same staff in a well managed herd with low levels of arthritis during the suckling period. The aim was to monitor the influence of floor type and bedding intensity on the incidence and severity of foot and skin lesions and development of arthritis in young piglets.

## Methods

### Herd, management routines and initial level of lameness

The study was carried out in a well managed conventional piglet producing herd with 180 Large White × Landrace sows. Pregnant sows were group housed in a deep-litter straw system, but fed individually. Every second week a batch of sows was transferred to a previously emptied and cleaned farrowing unit. These transfers were effectuated four days before the first sow of the group was expected to farrow.

The herd had four identical farrowing units with 15 identical farrowing pens housing loose sows. After farrowing, the canine teeth of the piglets were filed in litters with more than 12 piglets. Tail docking is forbidden in Sweden. Male piglets were castrated at three to five days of age, and at that time all piglets received an intramuscular injection of 200 mg iron as iron dextran (Pigeron: Leo Pharmaceutical, Copenhagen, Denmark). The piglets were offered a commercial creep feed without antibiotics from the age of ten days.

Piglets were weaned at an average age of 4.5 weeks, and records of diseases and medical treatments were kept for each sow and piglet. They were generally effectuated by the staff after instructions from the herd veterinarian. The mean incidence of treatments for lameness in piglets during the last two years prior to this experiment had been 4.1 ± 1.8% per farrowing batch.

The four farrowing units were identical and aged 10 years. The identical farrowing pens had a solid concrete floor and measured 7.0 m^2^, out of which 0.85 m^2 ^was a creep area exclusive to the piglets which was heated with infrared lamps and 2.6 m^2 ^was a drained plastic floor that filtered liquids but retained straw and faeces. The amount of chopped straw was visually judged as rich by the investigators. By repeated weightings before initiating the trial it was verified to correspond to 1 kg chopped straw per sow and 1 hg per piglet and day.

### Experimental groups and experimental animals

Nothing was changed in the management of the four identical farrowing units, but the surface of the floor was altered in two of them, and the amount of bedding was doubled in a third unit. Thus, four experimental groups were created:

**Group I **was left as a control and managed as prior to the initiation of this trial.

**Group II **aimed to evaluate the influence of increased amount of bedding material solely. The amount of chopped straw was doubled in comparison to the control group, *i.e*. 2 kg chopped straw per sow and 2 hg per piglet and day.

**Group III **aimed to evaluate effect of a commercial method used to repair the floor surface of concrete floors. Piglet Floor^® ^(Flowcrete Sweden AB, Perstorp, Sweden) is an epoxy free two-component solvent solution with natural quartz. The components were mixed and applied to the cleaned and dry concrete floors of the farrowing pens by certified professionals at a thickness of 2–4 mm. The amount of chopped straw used was equal to that of the control group, *i.e*. 1 kg chopped straw per sow and 1 hg per piglet and day.

**Group IV **aimed to evaluate effect of another commercial method used to repair the floor surface of concrete floors. Thorocrete SL^® ^(Växa Halland, Sweden) is a two-component product. Component 1 is a powder with cement, graded sand and additives. Component 2 is an acryl polymer emulsion. The two components were intermixed to a workable consistency and spread on the cleaned but still wet floors of the farrowing pens by certified professionals at a standard thickness of 4–5 mm. The amount of chopped straw used was equal to that of the control group, *i.e*. 1 kg chopped straw per sow and 1 hg per piglet and day.

### Animals

Three consecutive farrowing batches in each group were followed during the suckling period of 4.5 weeks. Thus, every batch born during a period of 24 weeks was included in the study. As earlier observations had revealed that the incidence of lameness in piglets not is influenced the parity number of the sow [8] we focused to create as large groups as possible that were born on the same day. **Group I **comprised 28 litters with 335 live born piglets, mean parity 4.5 ± 2.0 and a mean litter size 12.0 ± 1.6; **Group II **comprised 19 litters with 210 live born piglets, mean parity 5.1 ± 2.8 and a mean litter size 11.1 ± 2.4; **Group III **comprised 22 litters with 243 live born piglets, mean parity 5.1 ± 2.5;and a mean litter size 11.0 ± 2.3; and **Group IV **comprised 24 litters with 285 live born piglets, mean parity 3.3 ± 1.5 and a mean litter size 11.9 ± 1.6. Every litter was reared intact during the study period and here were no statistically significant differences in litter sizes between the groups.

### Lesions at feet, limbs and skin in piglets

The piglets born were studied until the age of 17 days with respect to presence of skin wounds and abrasions. A total of 1,073 live born piglets were individually examined day 3, day 10 and day 17. They were restrained and examined for presence of skin lesions at the carpus, hock, abdomen and teats, face and tail. The feet were examined with respect to presence of sole bruising. Sole bruising was defined as congestion and bruising of the solar corium presenting as a dark red pigmentation on the volar surface of the foot. The sex of each piglet was recorded and castration wounds were inspected. The severity of the lesions was scored and examined by using protocols previously described [3] as seen in table 1.

**Table 1:** The severity of the lesions recorded at day 3, day 10, day 17 was scored as 0, 1, 2 or 3 defined as shown below.

**Score**	**Skin lesions**	**Sole bruising/Sole erosion**	**Castration wounds**
**0 – No lesion**	-	-	-
**1 – Mild**	Hairless patches or loss of hair and mild hyperkeratosis	Smal part of the volar surface of digit affected	Mild inflammation, eczema or oedema
**2 – Moderate**	Skin abrasions	Less than half of the volar surface of the digit affected	Clinical signs of inflammation, swelling, redness, localized warmth
**3 – Severe**	Skin wounds. Spots of induration or scab that is a hard mass mainly of dried blood.	More than half of the volar surface digit affected or sole erosion, loss of horny tissue.	Inflamed castration wounds with purulence. Smelly wound. Abcess.

### Diagnose of arthritis

Lameness was registered individually until weaning. The herd veterinarian instructed and controlled medical treatments performed, but the technical staff generally administered them. Lameness and/or visibly swollen joint(s) were defined as arthritis, and affected piglets were treated parenterally with antibiotics.

### Confirmation of arthritis and antimicrobial susceptibility

Twenty seven of the 41 piglets diagnosed as lame before weaning were culled instead of medicinally treated. To exclude sprains, these piglets had to be lame and/or express visibly swollen joint(s) for 2 days before euthanized. They were stored at -20°C until necropsied when all limb joints were examined. Samples for bacteriology were collected with sterile cotton swabs from up to 3 affected joints and from a normal joint from each pig at necropsy. The samples were spread directly to blood agar (blood agar base No. 2; LabM, Salford, England + 5% horse blood) and bromcresol purple-lactose agar (NVI art No.341200). The plates were incubated at 37°C and read after 18 and 48 hours. Isolates of staphylococci, streptococci and *E. coli *were typed with methods used at the Bacteriological diagnostic laboratory at the National Veterinary Institute (NVI).

Isolates of staphylococci and streptococci were tested with respect to antimicrobial resistance towards penicillin, ampicillin, ceftiofur, spiramycin, neomycin, gentamicin, streptomycin, trimethoprim/sulfametoxazol, enrofloxacin, oxytetracycline, florfenicol and oxacillin (VetMIC™ Large Animal, NVI). Isolates of *E. coli *were tested for antimicrobial resistance towards ampicillin, ciprofloxacin, nalidixic acid, gentamicin, ceftiofur, streptomycin, tetracycline, florfenicol, kanamycin, sulfamethoxazole, trimethoprim, chloramphenicol and cefotaxime (VetMIC™ GN-mo, NVI).

### Statistical analyses

Data from the 1,073 liveborn piglets from four different floor types were included in the study. The prevalence of lesions at each inspection (day 3, day 10, day 17) was calculated as the number of piglets with that lesion divided with the number of piglets examined at that occasion.

Only the first time score for lameness in each piglet was taken into account; any recurrence of lameness was ignored in the analyses. The weekly risk incidence for treatment due to lameness was calculated as the number of piglets affected by lameness during the actual period divided by the number of live piglets previously not affected by lameness at the beginning of the period.

Data was statistically analysed using the SAS software ver. 9 [13]. Mean values of the piglets skin lesion scores and castration wounds (scores 0 to 3) were calculated for each group – sow – inspection day – sex – combination. The mean values were further analysed by analysis of variance using PROC MIXED. The statistical model included the fixed effects of group (4), day (3), sex (2) and the interaction between group and day. Also, the random effect of sow, nested within group was included in the statistical model. In the statistical model for analysing castration wounds, the effect of sex was excluded. Least-squares means were calculated for each level of the fixed effects, and pairwise tests of significance were performed using t-tests.

## Results

### Lesions at feet, limbs and skin in piglets

**Figure 1:**
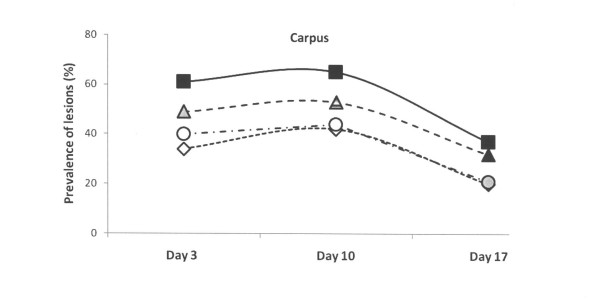
**Prevalence of skin lesions of piglets estimated on days 3, 10 and 17 and scored mild to severe at carpus in four experimental groups**. Rectangles represent Group I – control, triangles represent Group II – with doubled chopped straw, circles represent Group III – Piglet Floor^®^, and rhombs represent Group IV – Thorocrete SL^®^. Symbols other than black represent a statistic difference to black within examination day (stripe = p < 0.05; grey = p < 0.01; white = p < 0.001).

The skin lesions of the young piglets mostly consisted of hairless patches, abrasions or scabs. The skin lesions on the carpus and hock were nearly always bilateral and were observed as early as a few hours after farrowing. At 3 days of age, the prevalence of skin lesions at carpus ranged from 34% to 61% in the four groups (Figure 1). In comparison to the control group with abrasions in 61% of the piglets (n = 335), piglets born on the repaired floors had the lowest (p < 0.001) prevalences of abrasions at carpus (40% in Group III – Piglet Floor^®^, n = 243; 34% in Group IV – Thorocrete SL^®^, n = 285). Also the doubled straw ration decreased (p < 0.01) the abrasions (49% in group II, n = 210).

The skin lesions at carpus declined in all four groups from day 10 (Figure 1). On day 10 and day 17 of age, the prevalence of skin lesions at carpus in piglets born on the repaired floors (44% and 21% in Group III, 42% and 20% in Group IV) was significantly (p < 0.01 to 0.001) lower than in the control group (65% and 37%). At 10 days of age, also the group with the doubled straw ration (group II) had a lower (p < 0.05) incidence of skin lesions at carpus (53%) than the control group (Figure 1).

The relation between examined days and skin lesions at carpus shows a decreased intensity in lesion score with time, from moderate to mild lesions (Table 2). The healing between day 10 and 17 was mirrored by significantly (p < 0.001) decreased lesion scores in all four groups.

**Table 2:** Differences in least squares means within each housing system, and pairwise t-tests of significance for these differences.

	**Group I control**	**Group II double straw**	**Group III Piglet Floor^®^**	**Group IV Thorocrete SL^®^**
	
**Day 10 compared to Day 3**				
**Carpus**	-0.10^n.s.^	-0.00^n.s^	-0.03^n.s.^	0.04^n.s.^
**Hock**	-0.11***	-0.02^n.s.^	-0.14***	-0.06^n.s.^
**Abdomen&teats**	-0.08***	-0.12***	-0.10***	-0.05*
**Face**	0.12***	0.04^n.s.^	0.05^n.s^	-0.01^n.s.^
**Tail**	-0.01^n.s^	0.01^n.s.^	-0.02^n.s^	-0.03^n.s.^
**Feet**	-0.51***	-0.22**	-0.38***^.^	-0.23***
**Castration wounds**	-	-	-	-
				
**Day 17 compared to Day 10**				
**Carpus**	-0.48***	-0.33***	-0.33***	-0.31***
**Hock**	-0.03^n.s.^	-0.08*	0.01^n.s.^	-0.01^n.s.^
**Abdomen&teats**	-0.01^n.s.^	-0.03^n.s.^	-0.00^n.s^	-0.01^n.s.^
**Face**	-0.05^n.s.^	-0.07^n.s^	-0.05^n.s.^	-0.06^n.s^
**Tail**	-0.04^n.s^	-0.02^n.s.^	-0.01^n.s.^	-0.03^n.s^
**Feet**	-0.09^n.s.^	-0.11^n.s^	0.05^n.s.^	-0.06^n.s.^
**Castration wounds**	-0.05^n.s.^	-0.11*	0.06^n.s.^	-0.05^n.s.^

ns: not significant, *: p < 0.05, **: p < 0.01, ***: p < 0.001

**Figure 2:**
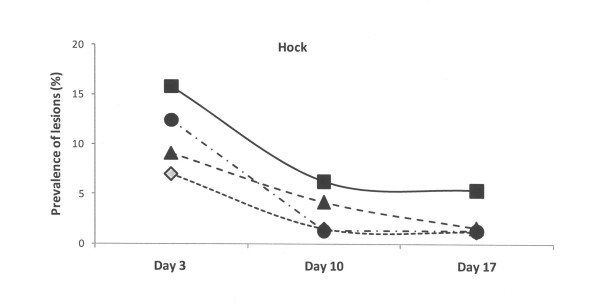
**Prevalence of skin lesions of piglets estimated on days 3, 10 and 17 and scored mild to severe at the hocks in four experimental groups**. Rectangles represent Group I – control, triangles represent Group II – with doubled chopped straw, circles represent Group III – Piglet Floor^®^, and rhombs represent Group IV – Thorocrete SL^®^. Symbols other than black represent a statistic difference to black within examination day (stripe = p < 0.05; grey = p < 0.01; white = p < 0.001).

**Figure 3:**
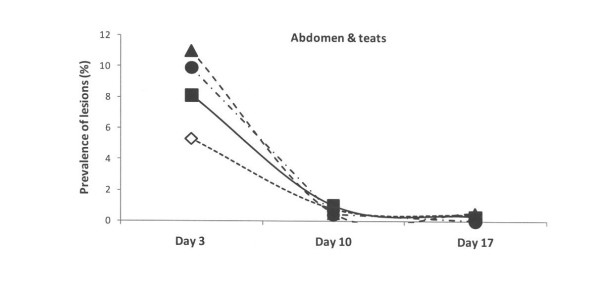
**Prevalence of skin lesions of piglets estimated on days 3, 10 and 17 and scored mild to severe at abdomen and teats in four experimental groups**. Rectangles represent Group I – control, triangles represent Group II – with doubled chopped straw, circles represent Group III – Piglet Floor^®^, and rhombs represent Group IV – Thorocrete SL^®^. Symbols other than black represent a statistic difference to black within examination day (stripe = p < 0.05; grey = p < 0.01; white = p < 0.001).

**Figure 4:**
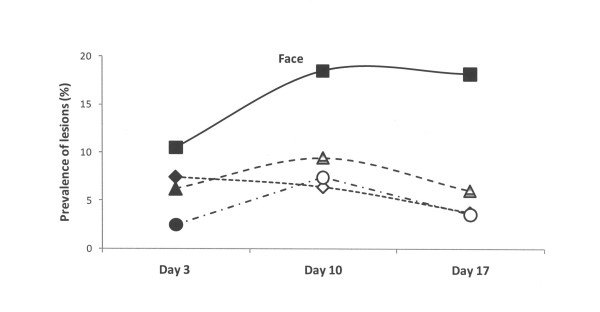
**Prevalence of skin lesions of piglets estimated on days 3, 10 and 17 and scored mild to severe in the face in four experimental groups**. Rectangles represent Group I – control, triangles represent Group II – with doubled chopped straw, circles represent Group III – Piglet Floor^®^, and rhombs represent Group IV – Thorocrete SL^®^. Symbols other than black represent a statistic difference to black within examination day (stripe = p < 0.05; grey = p < 0.01; white = p < 0.001).

**Figure 5:**
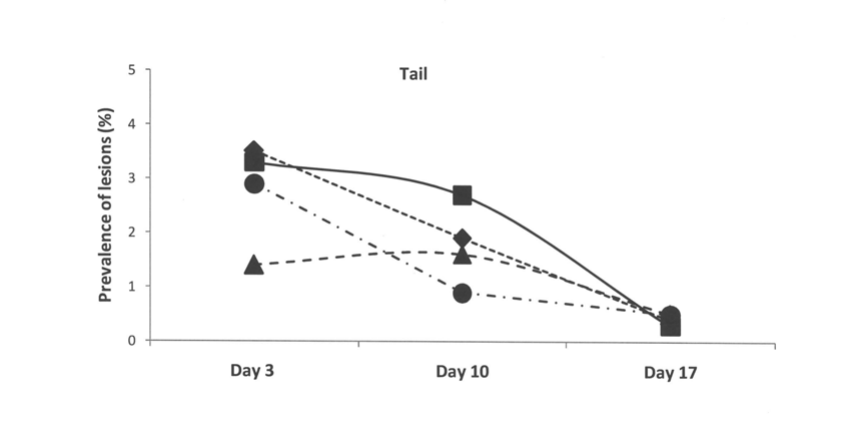
**Prevalence of skin lesions of piglets estimated on days 3, 10 and 17 and scored mild to severe at tail in four experimental groups**. Rectangles represent Group I – control, triangles represent Group II – with doubled chopped straw, circles represent Group III – Piglet Floor^®^, and rhombs represent Group IV – Thorocrete SL^®^. Symbols other than black represent a statistic difference to black within examination day (stripe = p < 0.05; grey = p < 0.01; white = p < 0.001).

**Figure 6:**
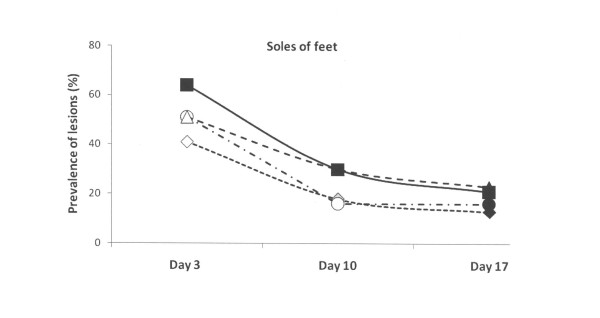
**Prevalence of sole bruising of feet of piglets estimated on days 3, 10 and 17 and scored mild to severe in four experimental groups**. Rectangles represent Group I – control, triangles represent Group II – with doubled chopped straw, circles represent Group III – Piglet Floor^®^, and rhombs represent Group IV – Thorocrete SL^®^. Symbols other than black represent a statistic difference to black within examination day (stripe = p < 0.05; grey = p < 0.01; white = p < 0.001).

For true percentages of affected pigs regardless of the score of the lesion, see figures 1 – 6.

At the third day of life, the lesions recorded at the other sites of the piglets (hocks, abdomen, face and tail) were of lower magnitude in comparison to those recorded at carpus in all four groups (Figures 2, 3, 4 and 5). The lesions recorded at hocks, abdomen and tails had practically vanished on day 10 and day 17 in all four groups. In contrast, the lesions in the face increased significantly in the control group and differed to the groups with repaired floors (p < 0.001) and the group with doubled straw ration (p < 0.05, Figure 4). For comparisons between groups and within groups over time, see also tables 2 and 3.

**Table 3:** Least-squares means for scores in each housing system and age, and pairwise t-tests between the groups.

**Day 3**							
	
**Group**	**Carpus**	**Hock**	**Abdomen&teats**	**Face**	**Tail**	**Feet**	**Castration wounds**
**Group I (Control)**	1.01	0.20	0.09	0.14	0.05	0.87	-
**Group II (Double straw)**	0.71	0.12	0.15	0.09	0.03	0.65	-
**Group III (Piglet Floor^®^)**	0.57	0.15	0.10	0.03	0.03	0.55	-
**Group IV (Thorocrete SL^®^)**	0.47	0.09	0.06	0.10	0.08	0.46	-
**Differences between groups**							
**Group II – Group I**	-0.30**	-0.08^**n.s.**^	0.06^**n.s.**^	-0.05^n.s^	-0.02^**n.s.**^	-0.22**	-
**Group III – Group I**	-0.44***	-0.05^**n.s.**^	0.01^**n.s.**^	-0.11^n.s^	-0.02^n.s^	-0.32***	-
**Group IV – Group I**	-0.54***	-0.11**	-0.03^**n.s.**^	-0.04^n.s^	0.03^**n.s.**^	-0.41***	-
**Group III – Group II**	-0.14^**n.s.**^	0.03^**n.s.**^	-0.05^**n.s.**^	-0.06^n.s^	0.00^**n.s.**^	-0.10^**n.s.**^	-
**Group IV – Group II**	-0.24**	-0.03^n.s^	-0.09***	0.01^n.s^	0.05^**n.s.**^	-0.19**	-
**Group IV – Group III**	-0.10^**n.s.**^	-0.06^**n.s.**^	-0.04^**n.s.**^	0.07^n.s^	0.05^n.s^	-0.01^**n.s.**^	-
**Day 10**							
	
**Group**	**Carpus**	**Hock**	**Abdomen&teats**	**Face**	**Tail**	**Feet**	**Castration wounds**
**Group I (Control)**	0.91	0.09	0.01	0.26	0.04	0.36	0.14
**Group II (Double straw)**	0.71	0.10	0.03	0.13	0.04	0.43	0.21
**Group III (Piglet Floor^®^)**	0.54	0.01	0.00	0.08	0.01	0.17	0.03
**Group IV (Thorocrete SL^®^)**	0.51	0.03	0.01	0.09	0.05	0.23	0.12
**Differences between groups**							
**Group II – Group I**	-0.21*	-0.01^n.s^	-0.02^n.s^	-0.13*	-0.00^**n.s.**^	0.07^**n.s.**^	0.08^n.s^
**Group III – Group I**	-0.37***	-0.08^n.s^	-0.01^n.s^	-0.17***	-0.03^n.s^	-0.19*	-0.11*
**Group IV – Group I**	-0.40***	-0.06^n.s^	0.00^n.s^	-0.17***	-0.01^**n.s.**^	-0.13*	-0.02^n.s^
**Group III – Group II**	-0.17^**n.s.**^	-0.09^n.s^	-0.03^n.s^	-0.05^n.s^	-0.03^n.s^	-0.26***	-0.18**
**Group IV – Group II**	-0.20	-0.07^n.s^	-0.02^n.s^	-0.04^n.s^	-0.01^**n.s.**^	-0.20**	-0.09^n.s^
**Group IV – Group III**	-0.03* ^**n.s.**^	0.02^n.s^	0.01^n.s^	0.01^**n.s.**^	0.04^n.s^	-0.06^**n.s.**^	0.09^n.s^
**Day 17**							
	
**Group**	**Carpus**	**Hock**	**Abdomen&teats**	**Face**	**Tail**	**Feet**	**Castration wounds**
**Group I (Control)**	0.43	0.06	0.00	0.21	0.00	0.27	0.09
**Group II (Double straw)**	0.38	0.02	0.00	0.06	0.02	0.32	0.10
**Group III (Piglet Floor^®^)**	0.21	0.02	0.00	0.03	0.00	0.22	0.09
**Group IV (Thorocrete SL^®^)**	0.20	0.02	0.00	0.03	0.02	0.17	0.07
**Differences between groups**							
**Group II – Group I**	-0.05^**n.s.**^	-0.04^**n.s.**^	-0.00^**n.s.**^	-0.15*	0.01^n.s^	0.05^**n.s.**^	0.01^n.s^
**Group III – Group I**	-0.22*	-0.04^**n.s.**^	-0.00^**n.s.**^	-0.18***	-0.00^n.s^	-0.05^**n.s.**^	-0.00^n.s^
**Group IV – Group I**	-0.23***	-0.04^**n.s.**^	-0.00^**n.s.**^	-0.18***	0.01^**n.s.**^	-0.10^**n.s.**^	-0.02^n.s^
**Group III – Group II**	-0.17^**n.s.**^	-0.00^**n.s.**^	-0.00^**n.s.**^	-0.03^n.s^	-0.01^n.s^	-0.10^**n.s.**^	-0.01^n.s^
**Group IV – Group II**	-0.18^**n.s.**^	-0.00^**n.s.**^	-0.00^**n.s.**^	-0.03^n.s^	-0.00^n.s^	-0.15^**n.s.**^	-0.03^n.s^
**Group IV – Group III**	-0.01^**n.s.**^	-0.00^**n.s.**^	-0.00^**n.s.**^	-0.00^**n.s.**^	0.01^n.s^	-0.05^**n.s.**^	-0.02^n.s^

ns: not significant; *: p < 0.05 **: p < 0.01; ***: p < 0.001

For true percentages of affected pigs regardless of the score of the lesion, see figures 1 – 6.

Also the sole bruising scores of the control group were higher than the other three groups at three days of age (p < 0.001). At day 10, sole bruising had decreased (p < 0.01–0.001, Table 2) within each group, but they were more commonly observed in the control group and the group with doubled straw ration, which both differed significantly (p < 0.01–0.001) from the groups with the repaired floors (Figure 6).

Healing castrations wounds were examined the 10^th ^and 17^th ^day of age. A low incidence of mild inflammations was recorded in all four groups (Table 3).

### Lameness and arthritis

**Figure 7:**
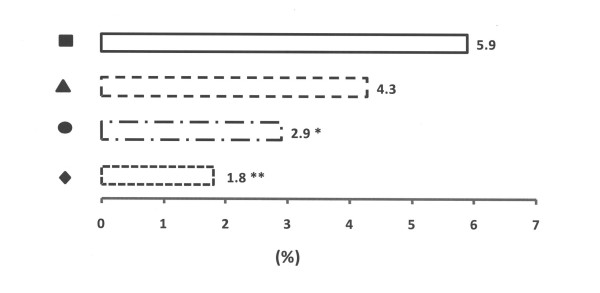
**The incidence of lameness during the first four weeks of life in piglets in four experimental groups**. Rectangle represents Group I – control, triangle represents Group II – with doubled chopped straw, circle represents Group III – Piglet Floor^®^, and rhomb represents Group IV – Thorocrete SL^® ^(* = p < 0.05; ** = p < 0.0; significant differences in relation to the prevalence for the Group I – control).

In total 41 piglets were diagnosed as lame, corresponding to 3.8% of all live-born piglets (n = 1,073), and 87.8% of these diagnoses took place during the first 3 weeks of life. The risk incidence of lameness decreased from 1.5% during the first week of life to 0.5% during the fourth week. The incidence of lameness was highest in the control group (5.9%) and lowest in the group with repaired floors (2.9%, p < 0.05, Group III – Piglet Floor^®^, and 1.8%, p < 0.01, Group IV – Thorocrete SL^®^, Figure 7).

### Confirmation of arthritis and antimicrobial susceptibility

Twenty seven randomly selected lame piglets before weaning were culled instead of medicinally treated and were necropsied to establish a diagnosis. Twenty of these piglets (6 from Group I; 5 from Group II; 6 from Group III and 3 from Group IV) were ensured a definitive diagnosis of infectious arthritis with demonstration of bacterial growth (subacute arthritis – 3 piglets; acute fibrin-purulent arthritis – 2 piglets; acute purulent arthritis – 14 piglets; chronic arthritis – 1 piglet). Fifteen of these piglets (75%) were affected by polyarthritis *i.e*. infection in more than one joint. Bacterial growth was never recorded in macroscopically healthy joints. The bacterial cultivations were dominated by *Streptococcus dysgalactiae *subsp. *equisimilis *(60%), but also included *Staphylococcus hyicus *subsp.*hyicus *(35%) and *Escherichia coli *(5%). No antimicrobial resistance was recorded. The isolates of *Streptococcus dysgalactiae *subsp. *equisimilis *and *Staphylococcus hyicus *subsp.*hyicus *isolates were sensitive to all antibiotics included in the VetMIC™ Large Animal panel, and the isolates of *E. coli *were sensitive to all antibiotics included in the VetMIC™ GN-mo panel.

## Discussion

Floors in farrowing crates present a dilemma because the needs of the sow differ from those of her piglets in a number of ways [14]. Piglets prefer floors with low abrasive properties [15-17]. Still, floor abrasiveness is a major cause of superficial injury in piglets in farrowing pens [18,19]. In contrast, the sow requires access to an abrasive surface in order to prevent hoof overgrowth. Indeed, attempts to provide a non-abrasive surface for piglets have resulted in floors that were too slippery for sows and gilts [20,21]. The results of this study show that reparation of the floor with the materials used decreased the significance of abrasions and sole bruisings. Also doubling the amount of chopped straw prevented development of skin lesions and sole bruisings to some extent.

The frequency of skin lesions increases markedly over the first 3 days of life [3,4,6,22]. The scab over the lesions will heal with time, and when the piglets are about 5 weeks old the healing process is normally completed [23]. In the first week of life the piglets spent most of their time lying in the nest [9]. If the heated nest area is hard and rough there is a risk that the skin lesions will become irritated which will delay the healing process [24]. This study showed a decreased intensity in skin lesion at carpus score with time, from moderate to mild lesions. The healing between day 10 and 17 was mirrored by significantly (p < 0.001) decreased lesion scores in all four groups. In contrast, the healing of the soles, *i. e*. the incidence of sole bruising, decreased significantly already between day 3 and 10 in all four groups. In agreement with previous reports [15,25] foot lesions developed very early in life, certainly because newborn piglets have extremely soft horn tissues on the soles, which become harder with age. Almost all piglets have previously been reported to develop sole bruising within the first 4 days of life with increasing severances between birth and 12 day of age, thereby also decreasing the activity including suckling [25]. In our study, the incidence of skin lesions at carpus and sole bruisings were lower in the systems with the repaired floors (Table 3, Figures 1 and 6).

Similar patterns, but with lower magnitudes were observed with respect abrasions over the hocks and skin lesions at faces, abdomens, teats and tails. These observations corresponded well to the distribution pattern of skin lesions previously reported by others [1,2,6,23,26].

Healing castrations wounds were examined at the 10^th ^and 17^th ^day of age. A low incidence of mild inflammations was recorded in all four groups. These observations in combination with an equal incidence of lameness with respect to sex suggest that castration not predispose to development of lameness, provided that they are effectuated skilfully and under aseptic conditions as also previously suggested [8].

Concrete floors can become very rough and abrasive which in turn may quickly remove horn and skin from the feet of newly born piglets causing acute lameness [12]. The results of this study suggest that the degree of roughness and abrasiveness of the floors contributes significantly to development of abrasions and sole bruising, and that proper maintenance of the floor can prevent that. The mean incidence of treatments for lameness in piglets during the last two years prior to this experiment had been 4.1 ± 1.8% which was significantly decreased in the groups with repaired floors (2.9%, p < 0.05, Group III – Piglet Floor^®^, and 1.8%, p < 0.01, Group IV – Thorocrete SL^®^). Also doubling the amount of straw decreased the incidence of abrasions, sole bruising and lameness. Thus, reparations of floor surface and amount of bedding material are important tools in preventing abrasions and lameness in suckling piglets. However, the floor Thorocrete SL^® ^was slippery for sows, which visualise the problems of creating a floor optimal for both sows and their offspring. Indeed, attempts to provide a non-abrasive surface for piglets have resulted in floors too slippery for sows and gilts [20]. Further, straw is not always effective in preventing skin lesions as the piglets may remove the litter out of the lying area with their physical activity [24,27].

Corresponding to earlier observations [3,4,8] around 75% of the treatment against lameness took place when the piglets were aged less than 3 weeks. Around 85% of these treatments took place during the first 3 weeks of life and the risk incidence of lameness decreased from 1.5% during the first week of life to 0.5% during the fourth week. The bacterial cultivations were dominated by *Streptococcus dysgalactiae *subsp. *equisimilis *(60%), a beta-hemolytic streptococci and member of the normal flora of the sow. The sows have therefore been considered to be the most important source of beta-hemolytic streptococci lesions in piglets [28]. Porcine neonates are particularly susceptible to streptococcal infection [29,30] and streptococci species are commonly isolated from piglets aged 1–3 weeks [31]. It may be argued that bacterial cultivations from frozen samples may not to be representative of the material at the time of sampling. However, death of bacteria by freezing is small at -17 to -30°C and below [32], and the results obtained by us also concur well with earlier reports [28-31].

Polyarthritis is a common problem in preweaned pigs [33] and approximately 18% of the litters and 3.3% of the pigs have been reported to be affected by polyarthritis within 4 days of age [34]. We recorded an overall lower level of piglets with arthritis at that age. Apart from the quality of the flooring and bedding, this probably also was dependant on the absence of tail docking and that teeth filing only was effectuated in large litters. Indeed, a lower incidence of polyarthritis in piglets have previously been reported from herds that do not have their piglets teeth clipped or tails docked [34,35]. Still, fifteen of 20 piglets (75%) with necropsy-confirmed arthritis were affected by polyarthritis *i.e*. infection in more than one joint, which indicate a hematogenic spread of environmental microbes that initially have infected the piglets through abrasions – confirming the wisdom words saying that minor wounds and poor friends not should be neglected.

## Competing interests

The authors declare that they have no competing interests.

## Authors' contributions

MZ and PW initiated the study and deigned it in co-operation with NL. EN effectuated the necropsies. MZ was the main investigator and head writer of the manuscript with help from the other authors. All authors read and approved the final manuscript.
